# Metabolic Pathways and Ion Channels Involved in Skeletal Muscle Atrophy: A Starting Point for Potential Therapeutic Strategies

**DOI:** 10.3390/cells11162566

**Published:** 2022-08-18

**Authors:** Ileana Canfora, Nancy Tarantino, Sabata Pierno

**Affiliations:** Section of Pharmacology, Department of Pharmacy and Drug Sciences, University of Bari, 70121 Bari, Italy

**Keywords:** skeletal muscle, atrophy, disuse, hindlimb unloading, sarcopenia, ion channels

## Abstract

Skeletal muscle tissue has the important function of supporting and defending the organism. It is the largest apparatus in the human body, and its function is important for contraction and movements. In addition, it is involved in the regulation of protein synthesis and degradation. In fact, inhibition of protein synthesis and/or activation of catabolism determines a pathological condition called muscle atrophy. Muscle atrophy is a reduction in muscle mass resulting in a partial or complete loss of function. It has been established that many physiopathological conditions can cause a reduction in muscle mass. Nevertheless, it is not well known the molecular mechanisms and signaling processes causing this dramatic event. There are multiple concomitant processes involved in muscle atrophy. In fact, the gene transcription of some factors, oxidative stress mechanisms, and the alteration of ion transport through specific ion channels may contribute to muscle function impairment. In this review, we focused on the molecular mechanisms responsible for muscle damage and potential drugs to be used to alleviate this disabling condition.

## 1. Introduction

Skeletal muscle is widely present in the entire body and is devoted to important functions, such as contraction, energy production, support to the skeleton, and gravity force neutralization. Due to different pathological conditions or changes in physiological function, this tissue may experience severe atrophy with a reduced number and area of fibers and a loss of mass and strength with consequently reduced activity. The most common causes of atrophy are due to prolonged physical inactivity, as occurs during immobilization, bed rest, and spaceflight, or are due to advanced age, malnutrition, denervation, and various pathological conditions. These conditions include cancer (cachexia), amyotrophic lateral sclerosis, AIDS, kidney failure, and diabetes [1,2]. Sometimes, atrophy may be a consequence of drug assumption (i.e., glucocorticoids). Atrophy may be the result of an alteration of catabolic and anabolic processes. In particular, reduced synthesis and excessive protein degradation may result in a severe form of muscle atrophy that can lead to disability and death.

Fast- and slow-twitch skeletal muscles are generally composed of a different proportion of the two main fiber types, the slow-twitch fibers (type I, oxidative) and the fast-twitch fibers (type II, glycolytic), which exhibit specific metabolic and contractile properties [2]. Atrophy may affect either the fast or slow fibers, but the slow-twitch muscles are mostly involved. A specific gene expression program may be activated in each muscle in response to atrophy [3]. In particular, atrogenes are thought to be important for protein loss. Muscle-specific RING finger protein-1 (MuRF1) is an E3 ubiquitin ligase selectively expressed in cardiac and skeletal muscles [4]. This protein is upregulated during skeletal muscle atrophy and is able to control sarcomere muscle protein degradation. This review investigates the mechanisms involved in skeletal muscle atrophy by focusing principally on the two main studied conditions such as disuse and sarcopenia. The understanding of the mechanisms able to regulate muscle mass will furnish the right knowledge on new therapeutic targets for the prevention and treatment of skeletal muscle atrophy.

## 2. Activation of Degradation Pathways in Skeletal Muscle Atrophy

### 2.1. The Ubiquitin-Proteasome System

**Figure 1:**
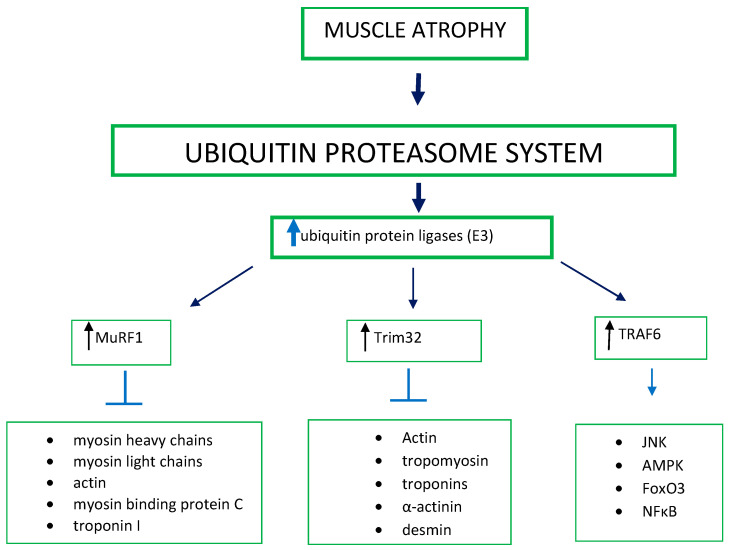
Ubiquitin-proteasome system and muscle atrophy. Principal mechanisms and different factors involved. The arrows up into the boxes indicate an increase of expression.

In skeletal muscle, the ubiquitin-proteasome system appears to play an important role in muscle loss. Indeed, a decrease in muscle mass may be associated with an upregulation of some ubiquitin-conjugating enzymes (E2) and some ubiquitin protein ligases (E3). The first muscle-specific ubiquitin ligases that were discovered to have a role in muscle loss were Atrogin-1/MAFbx and muscle ring finger-1 (MuRF1). Substrates of MuRF1 are myosin heavy chains, myosin light chains, actin, myosin binding protein C, and troponin I. It has been found that MuRF1 knockout mice are more resistant to muscle atrophy produced by different pathological conditions [5,6,7]. Accordingly, the MuRF1 overexpression in mice enhances ubiquitination of myofibrillar and sarcoplasmic proteins, and causes neuromuscular junction instability and muscle atrophy [6]. However, the absence of MuRF1 does not protect soleus muscle atrophy induced by spaceflight, showing that this model is quite different with respect to other models of atrophy [8]. During atrophy, other E3s are produced in smaller quantities, including Trim32, a ligase important for actin, tropomyosin, and troponins degradation [9]. Nevertheless, Trim32 knockout mice are sensitive to atrophy and show an inability to recover muscle mass after atrophy [10]. Another E3 ubiquitin ligase relevant to atrophy is TRAF6 [11], which promotes the union of Lys63-bound polyubiquitin chains to its target proteins. Muscle-specific TRAF6 knockout mice have low amounts of polyubiquitylated proteins in starving muscles [12]. TRAF6-mediated ubiquitylation is essential for the activation of different factors, such as JNK, AMPK, FoxO3, and NFκB [13], which, in turn, are important for atrogin-1 and MuRF1 expression (Figure 1).

### 2.2. The Calpain System

Another protein involved in muscle atrophy is calpain, which belongs to the calcium-dependent cysteine protease family [14]. Six isoforms have been identified in muscle, but only three have been identified by immunoblotting analysis [15,16]. Calpains are regulated by various factors, including calcium and phospholipids [16,17]. These proteins are generally localized at the level of the Z disk of the sarcomere [18], where they initiate the proteolytic cleavage of muscle proteins that make up the sarcomere [19,20]. Calpain has been shown to initiate in vitro the digestion of individual myofibrillar proteins, including desmin, filamen, protein C, tropomyosin, troponin T, troponin I, titin, nebulin, vimentin, gelsolin, and vinculin [21,22,23,24,25,26]. Due to its proteolytic properties, calpain appears to be involved in muscle atrophy. In vivo studies have also shown that in transgenic mice overexpressing calpastatin, a calpain inhibitor, the hindlimb unloading (HU)-induced atrophy was reduced by 30% [27]. Furthermore, the shift of myofibrillar myosin from slow-to-fast isoforms was avoided. These results show that calpain has an important role in muscle atrophy due to hindlimb suspension and sarcopenia [28,29]. Another study highlighted the role of calpain 1 in the ubiquitin-proteasome pathway and in the anabolic processes mediated by Akt-mTOR-p70S6K and MAPK-Erk (p90RSK) signaling. PD150606, a calpain inhibitor, was tested for its effect in three-day hindlimb-suspended (HS) rat muscle and compared with those found in HS untreated animals. The calpain inhibitor reduced the activation of calpain-1 mRNA expression, reduced activation of MAFbx mRNA, and reduced protein ubiquitination and eEF2 mRNA expression, again supporting the role of calpain in inducing muscle atrophy [30].

### 2.3. Autophagy-Lysosome System

Autophagy is a cellular catabolic process that warrants the degradation and recovery of cellular components. During this process, the damaged cytoplasmic elements are isolated from the rest of the cell within a membrane vesicle called the autophagosome. The membrane of the autophagosome fuses with that of a lysosome, and the inner contents are degraded and recycled.

Defects of autophagy have been observed in many infectious and autoimmune diseases, tumors, and neurodegenerative disorders. An important role of autophagy was discovered in maintaining muscle homeostasis: indeed, it can contribute to muscle degeneration, but it can also be a necessary mechanism for cell survival. For example, in Betlhlem’s myopathy and Ullrich’s congenital muscular dystrophy, two hereditary muscular disorders associated with collagen VI deficiency, autophagy has a protective role against muscle wasting since inhibition of the autophagic process determines a worsening of the disease [31,32]. In many vacuolar autophagic myopathies such as Pompe disease and Danon’s disease, it can be found an accumulation of autophagosomes due to an alteration of the lysosomal function [33]. The development of molecular and imaging tools has been useful for the characterization of autophagy in atrophied muscles [34]. Autophagy hyperactivation has been found to contribute to muscle loss in various conditions, such as cancer cachexia, fasting, sepsis, chemotherapy, disuse, and denervation [35,36]. Oxidative stress was generated by a mutant superoxide dismutase protein (SOD1-G93A) expression able to cause muscle atrophy by activating autophagy (in particular LC3, a protein that contributes to the formation autophagosome) in SOD1-G93A transgenic mice model of ALS [37,38], suggesting that the modulation of the autophagic process can be a promising therapeutic intervention to counteract muscle atrophy connected with oxidative stress.

Although few data are available regarding the role of autophagy in muscle atrophy, the inactivation of muscle-specific genes that codify for autophagy-related proteins demonstrates the function of autophagy in muscle homeostasis. For example, Atg7-null mice exhibit muscle atrophy. In fact, the removal of Atg7, an E1 enzyme of the autophagic process, causes activation of the unfolded protein response, which in turn triggers the degeneration of myofibers; this leads to inhibition of autophagosome formation, with consequent oxidative stress and accumulation of polyubiquitylated proteins [39]. Another example was observed in mice with muscle-specific removal of Atg5, a component of the autophagic mechanism [40]. Although there are few studies regarding autophagy in the skeletal muscle of elderly people, some studies have shown that abnormal regulation of autophagy could contribute to sarcopenia, which is a loss of muscle mass and function that occurs as a result of the aging process. A reduction in the autophagic progression and an increase in apoptotic mechanisms and oxidative stress with increasing age have been demonstrated in rat skeletal muscle. To confirm this, an autophagic stimulus in the muscles, such as calorie restriction, has been found to improve muscle condition during aging [41]. Activation of autophagy in skeletal muscle through FoxO avoids muscle weakness with increased lifespan [42].

### 2.4. Apoptosis Signaling Pathway

Apoptosis is responsible for the elimination of dysfunctional or damaged cells in several physiological and pathophysiological conditions. Apoptosis is caused by oxidative stress or DNA fragmentation [43] and leads to mitochondria dysfunction in terms of impaired membrane potential, reduced oxidative phosphorylation, and ATP production [35]. Mitochondrial apoptosis-associated atrophy can be the result of sarcopenia, disuse (i.e., immobilization and denervation), or specific myopathies (i.e., muscular dystrophy). The BCL2 family, involving pro- and anti-apoptotic proteins, plays a role in mitochondrial apoptotic signaling [44].

During muscle atrophy due to disuse (denervation, immobilization, hindlimb unloading), elevated total and mitochondrial BAX, altered total BCL2, and CASP9 activation are observed [45]. Importantly, Bax- and Bax/Bak-knockout attenuates muscle atrophy and a number of the mitochondrial apoptotic changes that occur with denervation [46]. Mechanistic studies in vitro demonstrated that elevation of miR-142a-5p suppressed mitofusin 1 (MFN1), leading to elevated total BAX:BCL2, CASP9, and CASP3 activation [47].

## 3. Anabolic Pathways Decline in Skeletal Muscle Atrophy

Muscle atrophy may also be caused by a decline in protein synthesis or impairment of regenerative pathways during disuse or other forms of atrophy [48]. This event can be associated with oxidative stress or with the alteration of the serine/threonine kinase, the mammalian target of rapamycin (mTOR), the principal regulator of protein synthesis by mediating the effects of growth factors [5,48,49,50,51,52]. Indeed, under disuse conditions, a decrease in protein synthesis is the result of mTOR inhibition. Accordingly, in adult rodents, a few days of treatment with rapamycin, a mTORC1 inhibitor, delays skeletal muscle growth [53]. In addition, a reduced translational ability after immobilization was observed in muscles of humans and animals [54,55].

Recent studies supported a role for UBR5, an E3 ubiquitin ligase, in the anabolic process and recovery from muscle atrophy; thus, the silencing of UBR5 results in skeletal muscle atrophy [56]. The knockdown of UBR5 was found to be associated with reductions in ERK1/2 and Akt phosphorylation, reduction in the level of eIF4e, which is involved in translation initiation, and consequent impairment of protein synthesis [56].

An anabolic role was discovered for beta-AR agonists [57]. Indeed, clenbuterol was found to accelerate the recovery from disuse-induced atrophy [58,59] and spinal and bulbar muscle atrophy [60]. The mechanism seems to involve the stimulation of protein synthesis through the enhancement of TORC1 activation [61,62]. However, hypertrophy is not always functional since a reduced normalized strength was observed [63]. In addition, nitric oxide (NO) was suggested to play a role in muscle hypertrophy via the activation of the transient receptor potential cation channel, subfamily V, member 1 (TRPV1), causing an increase in intracellular Ca^2+^ levels and an increase in protein synthesis through TORC1 activation by an unknown mechanism [64].

Recent studies highlight the possibility that the overexpression of a splice variant of PGC-1α, PGC-1α4, may induce muscle fiber hypertrophy through the downregulation of myostatin expression and increase in IGF-1 expression [65]. Importantly, PGC-1α4 expression was found to be increased in response to exercise [65].

Interestingly, skeletal muscle regeneration is impaired during disuse, likely due to decreased MyoD and myogenin expression and myostatin signaling upregulation [66,67].

## 4. Signaling Pathways in Skeletal Muscle Mass Loss

Skeletal muscle mass loss is one of the crucial symptoms of atrophy. Different factors are involved in the signaling pathways leading to atrophy. Nuclear factor kappa-light-chain-enhancer of activated B cells (NF-κB) is a protein complex that functions as a transcription factor. This plays an important role in regulating the immune response to infections, and its dysfunctions have been linked to cancer, inflammatory processes, autoimmune diseases, viral infections, and diseases of the immune system. NFκB is also expressed in skeletal muscle, where it mediates the effect of inflammatory cytokines, in particular tumor necrosis factor-α (TNFα), as observed during muscle loss and cachexia [68]. In the inactivated state, NF-KB is present in the cytosol, linked to an inhibitory protein. A variety of extracellular signals, including TNFα, can activate the IK B kinase (IKK) enzyme. IKK, in turn, is responsible for IκBα protein phosphorylation leading to its ubiquitination and degradation in the proteasome. Thus NF-κB is available. It has been found that NFκB activation in skeletal muscle determines muscle degeneration and inhibits regeneration in dystrophic muscles [69,70]. NF-κB can raise the expression of ubiquitin-proteasome system proteins (e.g., MURF1) and/or proinflammatory cytokines, and also chemokines able to contribute to skeletal muscle wasting. In fact, it has been observed that the muscle-specific overexpression of IKKβ in transgenic mice leads to muscle mass loss mediated by the action of the ubiquitin ligase E3 MuRF1 [71]. In support of this, it was found that the specific deletion of IKKβ in skeletal muscle in mice avoided the expression of the MuRF1 gene and consequently prevented atrophy. Despite the mechanisms still not being fully understood, NF-kB also seems to interfere with the IGF1-Akt signaling pathway. Indeed, it has been noted that conditional IKKβ knockout mice are resistant to muscle atrophy but demonstrate Akt hyper-phosphorylation [72]. Activation of NF-kB can also generate muscle atrophy by opposing myogenic differentiation. The “myogenesis” is the phenomenon that leads to the differentiation and formation of a skeletal muscle fiber, which starts during the first week of embryonic development. NF-κB inhibits myogenic differentiation through the accumulation of cyclin D1 and the activation of YinYang1 (YY1), a negative regulator of myogenesis, which works by blocking the synthesis of genes such as actin, creatine kinase, and myosin heavy chain IIb [73]. Activation of NF-κB has been shown to reduce levels of the MyoD protein [74]. MyoD, also known as myoblast-determining protein 1, is a myogenic transcription factor that plays an important role in regulating muscle differentiation. Activation of NF-κB increases the expression of the inducible nitric oxide synthase (iNOS) in muscle cells that sequester HuR (RNA-binding protein) by preventing transcription of MyoD [75]. Denervation-induced atrophy is related to the upregulation of specific histone deacetylases (HDAC), able to repress a negative regulator of myogenin, with an increase in MuRF1 expression and muscle wasting [76]. AMPK (protein kinase activated by 5′adenosine monophosphate) is also involved in muscle atrophy. Indeed, it has an important role in skeletal muscle metabolism and protein synthesis [77,78,79]. It is able to inhibit the anabolic processes that consume ATP [80] and regulate skeletal muscle mass. The inhibition of protein synthesis by AMPK is mediated by the mTORC1 block. It has been found that AMPK downregulation was able to attenuate atrophy during muscle disuse [81]. Interestingly, it has been found that a reduction in AMPK phosphorylation at the early stages of HU is likely related to reduced ATP consumption. After 3 days, the phosphorylation increases with increased activity. These events can drive many cellular processes in unloaded muscle, such as the proteolytic ones. However, at which time point muscle atrophy can rise and which factors are involved is still under study [82,83,84,85].

## 5. Oxidative Stress and Muscle Atrophy

Oxidative stress indicates a pathological condition caused by the failure of the physiological balance between production and elimination of oxidizing chemical species by antioxidant defense systems. It has been proposed to be the cause of muscle impairment in various muscle diseases, as it is one of the contributing factors to protein catabolism and, therefore, muscle atrophy and degradation [86]. In one study, the effects of oxidative stress were shown by comparing the slow-twitch soleus (Sol) and fast-twitch gastrocnemious (Gas) muscles after HU, a murine model of muscle disuse. Fast and slow muscles have adapted to disuse differently. In HU Sol, superoxide dismutase (Zn/CuSOD) expression is increased, peroxiredoxin-6 (PRDX6) and carbonic anhydrase 3 (CAH III) expression is reduced, thus underlining a weakening of the antioxidant defense systems. Although an increase in Zn/Cu SOD seems protective against oxidative stress, the reduced synthesis of enzymes downstream of Zn/Cu SOD, such as peroxiredoxin (PRDX6), can be responsible for hydrogen peroxide accumulation and thus for the oxidative stress increase [87]. Furthermore, the heat shock proteins (HSPs) that are able to protect cells from oxidative stress [88] in this situation were found to be downregulated. In addition, CAH III and PRDX6 are upregulated in Gas muscle during HU. Hence, no alteration of the antioxidant defense systems was observed in HU Gas. The protein oxidation index was much elevated in HU Sol than in CTRL Sol, while no differences were observed between HU Gas and CTRL Gas. These results highlighted an impairment of cellular defense systems during oxidative stress in HU Sol but not in HU Gas. In Sol, the administration of Trolox (an antioxidant compound) neutralized the increase in the oxidation index induced by disuse. Current findings indicate that oxidative stress is more likely to be a consequence of than a cause of muscle atrophy after HU in mice, both in a slow oxidative muscle, the Sol muscle, and in a fast glycolytic muscle, the Gas [89,90,91,92,93]. However, it is certain that oxidative stress plays a pivotal role in muscle disuse.

## 6. Muscle Atrophy Observed in Different Physiopathological Conditions and the Involvement of Sarcolemma Ion Channels

Muscle fibers can modify their phenotype according to demand by expressing diverse levels or forms of proteins essential in the control of muscle excitability. For example, a slow-to-fast phenotype transition occur following reduced muscular activity. This transition was observed during muscle disuse [94]. An accepted experimental rodent model of muscle disuse, the HU model, was used to simulate a condition of atrophy and to find possible therapies [95]. In this animal model, the slow-twitch Sol muscle exhibits severe atrophy already after 3 days of HU [96,97,98]. The soleus muscle is an antigravity muscle; thus, the reduction in gravitational loading leads to severe atrophy, muscle mass loss, and functional decline experienced as weakness, increased fatigability, and altered motor performance. In origin, muscle atrophy arises as an adaptive response of the body, with the aim of sustaining metabolic and energy homeostasis in these unfavorable conditions. Nevertheless, this response might turn into an adverse event with boosted muscle catabolism. A decrease in muscle fiber cross-sectional area (CSA) and an increase in mRNA encoding for the ubiquitin protein ligases, atrogin, and MuRF1, were found during the earliest days of disuse [99]. Together with muscle atrophy, we found a modification of the percentage of the various isoforms of the myosin heavy chain (MHC) in the soleus muscle. Accordingly, the expression and function of proteins involved in the control of muscle excitability, excitation–contraction coupling, energy metabolism, and contractile properties are changed toward those typical of the fast phenotype [98,100]. Indeed, the activity and expression of the ClC-1 muscle chloride channel that sustains the resting chloride conductance (gCl) were increased in HU rodents toward the value recorded in the fast muscles, being higher with respect to the slow-ones for physiological reasons. As already shown, gCl is pivotal for sarcolemma stability and for the regulation of the electrical and contractile properties. Thus, the HU Sol muscle loses its postural function and assumes characteristics of fast muscles [100,101]. Our previous studies have shown that after 3 days of HU, the increase in gCl in Sol is also accountable for a reduction in PKC activity and/or activation of a phosphatase, keeping the ClC-1 channels in an active state [100]. We found that the modification of gCl in the HU Sol muscle precedes the modification of the amount of MHC isoforms, proposing a role of this parameter in promoting the phenotype transition [98,102]. Accordingly, Sharlo et al. (2019) [103] did not find significant changes in MyHC type I or fast MyHC fibers at 3-days of unloading. In addition, the PGC-1α transcript and protein were unchanged during the same period [104]. However, it should be underlined that, in contrast to our results, other authors have measured a modification of the MHC already at 24 h [83,105,106,107,108]. It can be hypothesized that after an acute, immediate effect (24 h), it is possible that at 3 days of HU, the modification was attenuated or subjected to a temporary balance between degradation and compensative synthesis under a specific nerve stimulus. Afterward, the slow-to-fast transition and the modification of MHC become stable.

Numerous studies indicate that insulin can modulate the expression of PKC [109]. It would therefore be useful to assess the possible link between PKC expression and/or activity and insulin resistance in models of reduced motor activity, such as the HU rodent. Thus, a possible therapeutical approach able to modulate the activity of PKC can be suitable to reduce the damage induced by muscle disuse.

Very little is known about the mechanism that regulates the expression of ClC1. It would appear that the expression of the ClC1 channel is under nerve control. There are two regulatory pathways proposed: the pathway involving MyoD, belonging to the family of helix-loop-helix transcription factors, and the NFAT signaling pathway involving calcium-dependent calcineurin [110,111]. Between the two, only the first way seems to be involved during the HU. This increase in gCl is closely related to an increase in the activity of ClC1 channels and consequently results in a slow-to-fast transition of the muscle fibers type. It is possible to prevent muscle impairment and motor disability after space flight by pharmacologically modifying the gCl in muscle fibers [94].

Chloride channels seem not to be the only ones involved in muscle damage. Indeed, compared to healthy rodent Sol muscle, a lower cytosolic calcium level is presentin the HU Sol muscle supporting the slow-to-fast phenotype transition [112,113]. The studies of Ingalls et al. [114,115] have shown an increase in the resting Ca^2+^ level in the Sol muscle of HU mice in the advanced phases of HU. This increase occurs after 2 weeks of HU and suggests protease system activation related to deep atrophy. The increase in calcium in the sarcoplasm involves L-type Ca+ channels and sarcoplasmic reticulum ryanodine receptors. The accumulation of calcium promotes Ca^2+^-dependent proteases, calpains, and contractile protein degradation [116,117,118,119,120].

Moreover, the immunoblotting technique shows the involvement of other channels. An increase in aquaporin-4 (AQP4) water channel in Sol muscle was found during simulated microgravity in response to muscle atrophy. This effect parallels the HU-induced slow-to-fast twitch conversion [97]. 

Important modifications are also observed in the activity and expression of the ATP-sensitive potassium channels, named K(ATP), during atrophy in Sol muscle, together with a decrease in fiber diameter [121]. Non-atrophic fibers MHC-IIa had higher K(ATP) currents with respect to the atrophic fibers. Fast-twitch muscles were not affected by atrophy in 14-day HU rats, and this is associated with the higher expression and activity of Kir6.2/SUR1 subunits of the K(ATP) channels, which characterize the fast muscle phenotype. The in vitro application of glibenclamide, a K(ATP) channel blocker, reduced the K(ATP) currents, and this effect is associated with atrophy. In support, muscle atrophy was prevented by the K(ATP) channel opener diazoxide [121]. Sodium channels also play an important role. It is known that sodium channels are crucial in the genesis of the action potential in excitable cells. In skeletal muscle, while fast-twitch fibers require a high density of sodium channels to obtain a fast and strong contraction, slow-twitch muscle fibers do not require a high density of channels. A lower density of sodium channels, in fact, can reduce the tendency to fatigue after exertion. This is probably related to the difficulty of the Na^+^/K ATPase pump to remove Na^+^ from the sarcoplasm in conditions of sodium excess. On the other hand, situations of difficulty in maintaining posture and reduced motor capacity after a space flight or after a period of immobilization can be explained by an increase in sodium density not compensated by an increase in the activity of sodium potassium ATPase [122]. Pharmacological block of sodium channels in slow-twitch muscle fibers could be a helpful approach in counteracting muscle damage [122]. It has also been found that muscle disuse also determines changes in the alpha 2 isoform of Na, K ATPase. Na, K ATPase is important in the excitability and contractility of skeletal muscle [123,124]. One study examined the alpha 1 and alpha 2 Na, K ATPase isoforms using a six-hour hind limb disuse animal model. The activity of Na, K ATPase after six hours was reduced in the soleus muscles at rest due to a reduction in enzymatic activity and accompanied by resting membrane potential depolarization. Remarkably, muscle workload restores resting potential and Na, K ATPase function [125]. Accordingly, other studies [98,122,126,127] show that after 3–7 days of suspension of the limbs, the soleus muscle shows a decrease in the resting membrane potential, deriving from the reduced electrogenic activity of alpha 2 Na, K ATPase. To deepen the mechanism, it has been tested the effect of ouabain at subnanomolar concentrations. This compound activates the Na, K ATPase and hyperpolarizes the sarcolemma [128]. Thus, ouabain can prevent sarcolemma depolarization and the disuse-induced dysfunction in rodent soleus muscle [129].

Recent studies have highlighted that the Ca^2+^-associated-Piezo 1 channel is involved in muscle atrophy. Piezo 1 is in charge of maintaining constant calcium levels in skeletal muscle; thus, inhibition of Piezo 1 causes muscle atrophy through a signaling pathway involving the transcription factor Krüppel-like factor 15 (KLF15) and the cytokine interleukin-6 (IL-6). In addition, a reduced expression of Piezo 1 was observed in skeletal muscle biopsies of patients undergoing cast fixation after bone fracture and then immobilization with respect to control subjects [130]. In addition, it was shown that in mice in which the hind limbs were immobilized for three days, an increase in KLF15 was seen. In contrast, in mice lacking KLF15 (M-KLF15KO mice), atrophy of skeletal muscle caused by immobilization was prevented. In addition, in these M-KLF15KO mice, it was found that IL6 expression was also prevented. Finally, chromatin immunoprecipitation analysis showed how KLF15 binds to the promoter region of the Il6 gene. The relationship between Piezo 1, KLF15, and IL6 was clearly assessed by treating primary mouse myofibers or C2C12 myotubes with GsMTx-4, a pharmacological inhibitor of Piezo1; this compound was able to increase KLF15 and IL6 levels [124]. Interestingly, KLF15 mRNA was increased by glucocorticoids, thus triggering muscle atrophy [131,132].

A severe form of atrophy was due to sarcopenia, an age-related loss of skeletal muscle mass associated with functional disability [133]. A loss of muscle proteins resulting from a disproportion between protein synthesis and breakdown occurs [134]. In this particular condition, atrophy is accompanied by a loss of fast-twitch fibers [135]. Our previous findings have demonstrated important modification of the activity of skeletal muscle protein during aging [102,136,137] associated with increased ROS levels and a decrease in the protective process. A downregulation of the expression of ClC-1 protein, linked with an abnormal decrease in the resting chloride conductance (gCl), was observed in the fast-twitch EDL muscle [138,139]. This channel is a key determinant of muscle excitability; indeed, a marked reduction in gCl is characteristic of disabling syndromes such as Myotonia Congenita [140]. Thus, this reduction can justify the development of skeletal muscle symptoms complained by elderly subjects, such as myalgia and muscle weakness. The activity of ClC-1 is regulated by protein kinase C (PKC), which activation is responsible for channel closure [141]. This regulation is modified during aging and contributes to gCl reduction [142]. 

**Figure 2:**
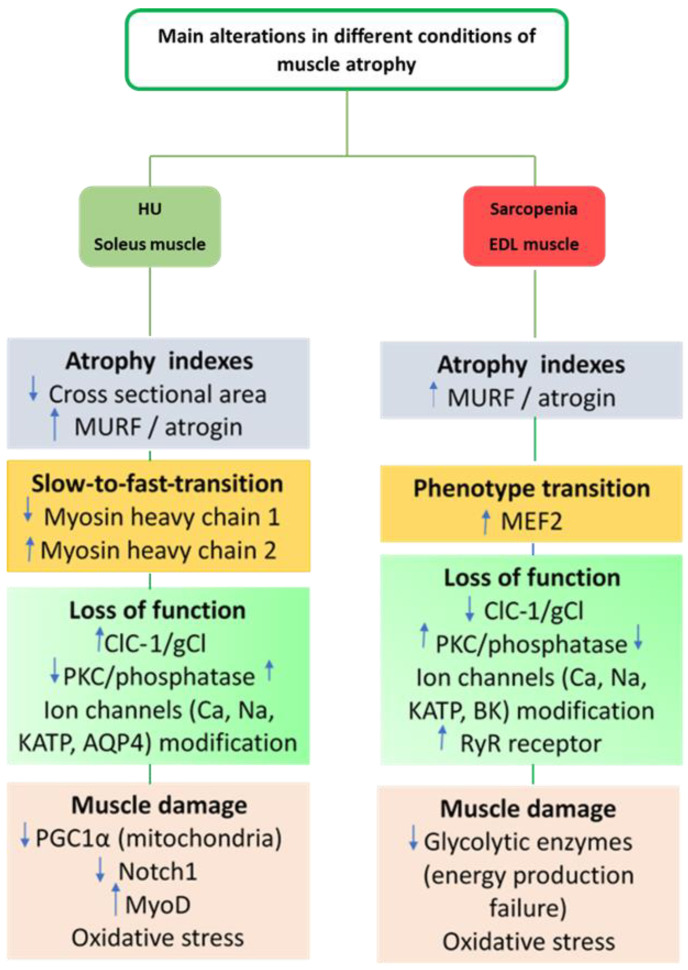
A brief description of the main mechanisms contributing to muscle atrophy during HU or aging in different muscle types. Soleus muscle as a slow-twitch muscle. Extensor digitorum longus (EDL) as a fast-twitch muscle. Arrows up indicate an increase and arrows down a decrease.

In a preclinical model of cisplatin-induced cachexia, severe atrophy has been found in terms of cross-sectional area (CSA) decrease, and MURF-1 increased expression associated with a significant increase in resting intracellular calcium and functional modifications. Indeed, a decrease in membrane ionic conductances with an increase in sarcolemma excitability was found in EDL muscle [143]. A brief description of the main mechanisms contributing to muscle atrophy during HU or aging is shown in Figure 2.

## 7. Glucocorticoid-Induced Skeletal Muscle Atrophy

**Figure 3:**
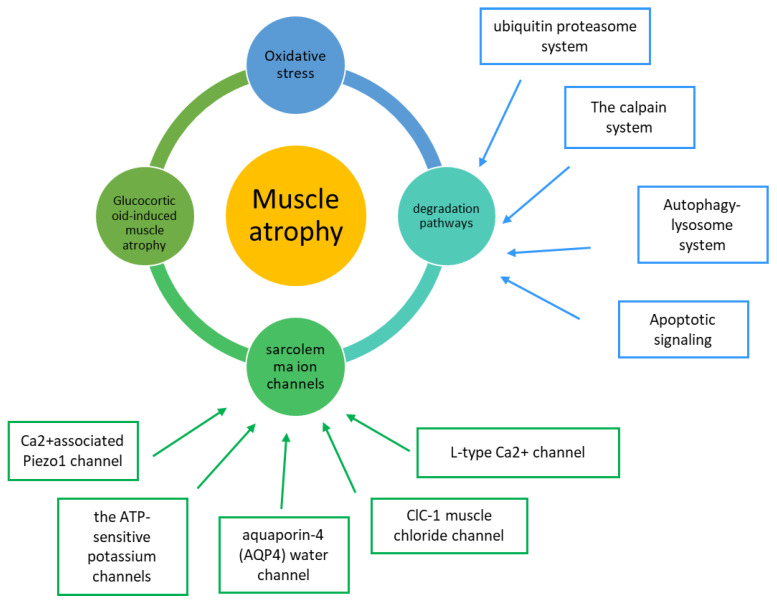
Summary of the principal mechanisms leading to muscle atrophy. Focus on degradation pathway and ion channel involvement.

Both endogenous and exogenous glucocorticoids may negatively affect the preservation of muscle mass and function. Glucocorticoid overflow can contribute to muscle atrophy [144]. Glucocorticoid-induced muscle atrophy relies on an increase in protein catabolism and reduced protein synthesis. The action of glucocorticoids on protein degradation is mediated by atrogenes such as FOXO, Atrogin-1, and MuRF-1. In particular, the myosin heavy chain contractile proteins are the most affected [145]. It is thought that the inhibitory action of glucocorticoids on muscle protein synthesis derives mostly from the inhibition of the mTOR pathway. The glucocorticoid receptor promotes the expression of two genes, codifying for REDD1 and KLF15 [146]. REDD1 inhibits the activity of mTOR. Inhibition of mTOR activates KLF15, promoting atrophy. KLEF15 is a transcription factor involved in several metabolic processes in skeletal muscle, for example, in the upregulation of branched-chain aminotransferase 2 (BCAT2), which, in turn, induces the degradation of branched-chain amino acids. Furthermore, KLF15 contributes to muscle catabolism through the transcriptional regulation of FoxO1, atrogin-1, and MuRF1 (Figure 3). 

## 8. Potential Drugs and Natural Compounds as Pharmacological Countermeasures That Can Improve Muscle Atrophy

Although numerous therapeutic targets have been identified to counteract muscle loss and possibly attenuate or improve muscle atrophy, there are few approved therapies. The most studied targets belong to the anabolic pathways and to the ubiquitin-proteasome system. For example, the inhibition of some specific muscle proteins such as MAFbx or Foxo, ATF4, Gadd45a, p21, and MEKK4 transcription factors can avoid the loss of muscle mass without leading to a hypertrophic condition. Among the different ubiquitin ligases, MuRF1 and TRAF6 have been studied for the development of muscle-specific inhibitors. However, the removal of MuRF1 or TRAF6 only partially preserves muscle atrophy during denervation in mice [5,130]. As an example, MG132 proteasome inhibitor can prevent muscle atrophy associated with muscle disuse [147]. In addition, the commercially available bortezomib has been found to be effective in the recovery of cisplatin-induced muscle mass loss in mice [148].

Apart from the inhibitors, anabolic therapies to mitigate disuse atrophy are expected to be more effective than therapies aimed to contrast protein degradation [1,149]. Several agents, such as anabolic or androgenic steroids, selective androgenic receptor modulators, growth hormone and analogs, myostatin inhibitors, β-receptor agonists, and nutritional food, are suggested and have been shown to induce variable efficacy. 

Bimagrumab, a monoclonal antibody against the activin II receptor that belongs to a TGF-beta receptor family, also merits particular attention. It increases muscle volume, lean muscle mass, and functional status in some muscle disuse conditions [150]. 

### 8.1. IGF1 and Its Analogs as Anabolic Compounds to Improve Muscle Damage

**Figure 4:**
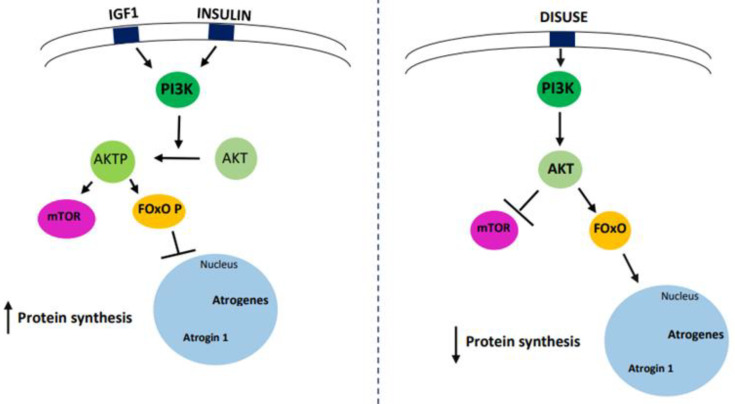
Insulin and IGF1 activate the PI3K-AKT-mTOR pathway, resulting in protein synthesis and hypertrophy. On the other hand, disuse condition leads to a blockage of the AKT signaling pathway. The arrows indicates an increase of protein synthesis in normal condition or a reduction of protein synthesis during disuse.

Some studies conducted on animals and humans have highlighted the pivotal role of the IGF1 hormone in muscle disuse and other atrophic conditions [1]. IGF1 is a hormone produced mainly by the liver and skeletal muscle. It promotes bone growth and muscle mass increase through the activation of specific signaling pathways. It has been found that IGF1 overexpression or chronic treatment can favor muscle hypertrophy and can induce recovery from muscle damage in animal models of neuromuscular diseases. Thus, exogenous IGF-1 may have therapeutic properties in the treatment of degenerative muscle diseases, sarcopenia, and other forms of skeletal muscle atrophy [151]. IGF 1 appears to be able to improve the condition of muscle disuse through the regulation of protein synthesis or the control of post-transcriptional mechanisms [152]. Among the different isoforms of IGF1, IGF-1Ea is the one capable of increasing strength and muscle mass. Insulin and IGF1 have been shown to activate the PI3K-AKT-mTOR pathways, fundamental in hypertrophy (Figure 4). Insulin/IGF1 signaling interferes with the mTOR kinase, which, in turn, cooperates with different proteins to form two different complexes: the rapamycin-sensitive TORC1 complex, which includes Raptor, and the rapamycin-insensitive TORC2 complex, which includes Rictor. While the Rictor knockout mice in the muscle do not exhibit an evident phenotype, Raptor and mTOR-deficient mice show decreased growth of fast but not of slow muscle fibers. In addition, overexpression of IGF-1 has been shown to have limited effects in preventing HU-induced muscle atrophy since, after HU, slow or fast muscles are no longer responsive to the hypertrophic action of IGF-1. However, it has been observed that the increase in gCl during HU in Sol muscle is counteracted by overexpression of IGF1, thus playing an important role in the phenotype transition [102] (Figure 4).

### 8.2. Other Anabolic Compounds Are Able to Improve Muscle Atrophy

Several studies have highlighted the use of anabolic steroids, such as testosterone and its analog nandrolone (ND), to counteract muscle atrophy in various conditions of disuse and diseases. ND supplementation has shown an increase in muscle mass in HIV-infected subjects or in hemodialysis patients. In rodents, ND improves muscle strength in the mouse model of Duchenne muscular dystrophy [153]. In the HU rats, ND is able to counteract HU-induced Sol muscle weight loss. This anabolic effect is mediated by its binding to the androgen receptor (AR), which leads to the activation of specific DNA sequences and affects the transcriptional activity of specific anabolic genes. The anabolic effect can induce an increase in protein synthesis with a hypertrophic effect [154]. In a previous study, mice were pre-treated for 2 weeks before HU and continued for the entire duration of the HU period (4 weeks of total administration). In this condition, ND had a beneficial effect on protein synthesis. The eIF2 protein (eukaryotic initiation factor of protein translation) was decreased during HU and restored in HU-ND mice. The ND treatment prevented the alteration in MyoD and Notch-1 expression suggesting the advancement of myogenic stem cell differentiation in response to atrophy. However, despite its action at the protein level, Sol muscle function was not restored after ND treatment. In addition, ND was not able to modify gCl and restCa or reverse the slow-to-fast phenotypic transition [94]. Myostatin, a protein discovered during studies on cell differentiation and proliferation, inhibits muscle development. It is produced by skeletal muscle cells, and its activity is regulated by the presence of an inhibitor called follistatin [155]. Myostatin has aroused particular interest among scientists in the study of myostatin inhibitors. However, myostatin inhibitors have shown important side effects in humans [156]. Interestingly, beta-2-adrenergic agonists, including clenbuterol or formoterol, have been identified as possible anti-atrophic drugs. These drugs promote protein synthesis and inhibit its degradation. Most of the effects of clenbuterol are mediated by the Akt-mTOR signaling pathway [157]. However, according to some studies, the action of β2-adrenergic agonists is expressed through the inhibition of glycogen synthase kinase-3 beta (GSK-3β) and the activation of another kinase, such as the p70S6K1. Although they are very effective, these drugs are modestly used due to the possible side effects that could occur in the cardiac tissue.

Another interesting compound is ghrelin, a stomach-releasing peptide that stimulates the appetite and growth hormone secretion. It appears to play an important role in the treatment of sarcopenia and other atrophy conditions. In fact, in one study, 20-month-old male WT and Ghrl ^−/−^ mice were used and fasted for 48 h. After 48 h, the muscle mass of the tibialis muscle was similar in the WT and Ghrl mice, while the mass of the gastrocnemius muscle was significantly reduced in the Ghrl ^−/−^ mice compared to the WT mice. Ghrelin null mice (Ghrl ^−/−^) are more prone to fasting-induced muscle atrophy, particularly in the Gas muscle. In these mice, there is also a reduced expression of the myogenic regulator MyoD and a higher expression of the protein degradation marker MuRF1 [158].

It is also interesting the role of S1P (sphingosine 1 phosphate), a signaling sphingolipid, defined as a bioactive lipid mediator. S1P is involved in both physiological and pathological processes [159,160]. Furthermore, it has an important role in the activation and proliferation of satellite cells [161,162]. Indeed, S1P plays an important role in skeletal muscle [163] regeneration in injured mdx mice [164]. In S1P null mice, the contraction tension of the EDL muscle was smaller with respect to that of the WT [165].

Recent studies have reported that losartan, an angiotensin 1 receptor antagonist, was able to partially protect soleus muscle from hindlimb unloading-induced atrophy, likely through a decreased TGF-β signaling and inhibition of remodeling [166].

**Figure 5:**
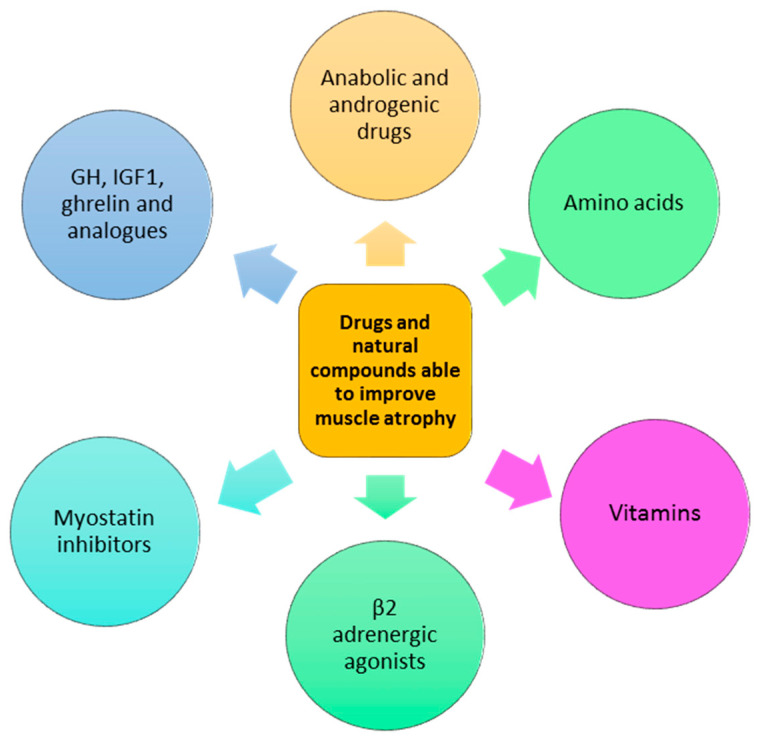
Drugs and natural compounds able to improve muscle atrophy.

The most therapeutical approaches are shown in Figure 5 and Table 1.

### 8.3. Natural Compounds Are Effective for the Recovery of Muscle Mass

In addition to drugs, particular attention has been devoted to natural compounds for the prevention of skeletal muscle mass loss and/or for muscle function recovery [167]. As previously reported, muscle atrophy is characterized by an increase in protein catabolism or reduction in anabolic processes. Therefore, the administration of protein supplements or compounds able to stimulate protein synthesis could be useful in the prevention of atrophy. For instance, ursolic acid stimulates muscle hypertrophy and reduces the expression of MuRF-1 [167] in soleus and extensor digitorum longus (EDL) muscle of chronic kidney disease mice. It also reduced myostatin and insulin-like growth factor-binding protein 3 (IGFBP3) in dexamethasone-treated C2C12 cell lines [168]. Apigenin was found to increase protein expression and skeletal muscle mass [169]. It is also a potent ROS scavenger [167].

Leucine is an essential amino acid able to increase muscle protein synthesis in C2C12 myotubes by activating the mTOR complex and the 4E binding protein-1 (4E-BP1) signaling pathway. This is an intracellular pathway that controls muscle protein synthesis. Furthermore, physical exercise together with the administration of a mixture of essential amino acids (L-isoleucine, L-histidine, L-leucine, L-lysine, L-methionine, L-phenylalanine, L-threonine, and L-valine) have been shown to avoid the loss of muscle mass in humans during 28 days of bed rest [170].

D-methionine, another essential amino acid found in some aliments such as yogurt, cheese, meat, and eggs, increases soleus and gastrocnemius muscle weight in the cisplatin-induced rat muscle atrophy model. The action of D-methionine is due to the downregulation of MAFbx and MuRF1 and to the upregulation of myogenin and MyoD [171].

Taurine is a sulfonic amino acid prevalent in excitable tissues and of fundamental importance in the function of skeletal muscle [172,173]. Taurine controls muscle excitability directly or mediated by PKC and modulates intracellular calcium homeostasis. Some studies have shown that muscle disuse induces a reduction in the taurine content in postural muscles [172]. The oral administration of taurine in HU rats modulated some muscle parameters involved in excitability and excitation–contraction coupling. In fact, it preserved resting gCl and resting cytosolic calcium concentration (restCa), corrected permeability of the calcium membrane through the sarcolemma cation channels, and improved the phenotypic transition induced by HU. In addition, taurine restored the expression of MURF-1, suggesting an early protective effect of this amino acid in the attenuation of the atrophic process [172,174]. Some studies have shown that in C2C12 myotubes, taurine avoids muscle atrophy induced by cisplatin by restoring microtubular and mitochondrial function and reducing the overload and localization of autophagolysosomes [175]. 

Branched-chain amino acids (BCAAs: leucine, isoleucine, valine) supplements were found to be beneficial for counteracting muscle atrophy in the HU rodent model due to their anabolic properties. Indeed, they can assist peptide synthesis and prevent protein breakdown. The parameters ameliorated by BCAAs action were myofiber CSA, muscle force, and contraction, as well as protein synthesis mediated by the mTOR pathway [176,177]. Accordingly, BCAA supplementation in older people has been found to improve muscle mass and strength [178]. Creatine is an energetic compound able to induce the recycling of adenosine triphosphate (ATP) in skeletal muscle tissue. It can have a beneficial role during immobilization-induced muscle disuse in humans [179,180]. Alongside amino acids, vitamins also play an essential role in the prevention of muscle atrophy characterized by an increase in oxidative stress and a decrease in the production of endogenous antioxidant species. The production of free radicals consequently determines an increase in protein catabolism. For example, vitamin C deficiency has been shown to cause muscle atrophy in mice, associated with high expression of MAFbx and MuRF1 and hyperproduction of ROS [181]. Vitamin E has been described as an antioxidant able to prevent hindlimb immobilization-induced muscle atrophy in rats [182]. One study found that oral vitamin D and E supplementation reduced muscle mass loss and improved quality of life in sarcopenic individuals [183].

**Table 1:** Drugs and compounds with pharmacological action able to prevent or restore muscle atrophy in different physiopathological situations.

Pharmaceutical Compounds That Could Prevent or Restore Muscle Atrophy		
Effect	Drug	Mechanism of Action
Inhibition of catabolic pathways	Calpastatin PD150606 L-arginine	Calpain inhibitor [27] Calpain inhibitor [30] nNOS activation and calpain inhibition [30]
Inhibition of catabolic pathways	Follistatin	Myostatin inhibitor [155]
Inhibition of catabolic pathways	Bortezomib	Proteasome inhibitor [148]
Upregulation of anabolic pathway	Losartan	TGF-β signaling inhibitor [166]
Upregulation of Anabolic pathway	β2-adrenoreceptor agonists	Akt-mTOR signaling pathway amelioration [157]
Upregulation of Anabolic pathway	Bimagrumab	Monoclonal antibody against activin II receptor [150]
Upregulation of Anabolic pathway	Nandrolone	Protein synthesis [94]
Upregulation of Anabolic pathway	Ghrelin	Growth hormone secretion stimulation [158]
Upregulation of Anabolic pathway	Insulin-like growth factor (IGF)-1 and analogs	Myoblast proliferation [155]

## 9. Conclusions

In this review, we summarized the mechanisms leading to muscle atrophy in different pathophysiological conditions with the aim of identifying effective countermeasures. Different drugs and natural compounds have been described. In this regard, it is important to underline that different clinical trials have been recruited to evaluate the efficacy of several pharmacological approaches in the different atrophy conditions For muscle atrophy due to disuse, the effects of leucine supplementation and anti-myostatin drugs have been studied. For sarcopenia, clinical trials reported different compounds, such as creatine, testosterone, bimagrumab, BCAA, androgen receptor modulators (SARM), losartan, citrulline, and IGF1 have been tested. In addition, different compounds have been tested for cancer cachexia atrophy, among which: androgen receptor modulators, bimagrumab, ghrelin, and growth hormone secretagogue receptor synthetic agonists.

## Data Availability

Not applicable.
